# Microbiota-Liver-Bile Salts Axis, a Novel Mechanism Involved in the Contrasting Effects of Sodium Selenite and Selenium-Nanoparticle Supplementation on Adipose Tissue Development in Adolescent Rats

**DOI:** 10.3390/antiox12051123

**Published:** 2023-05-19

**Authors:** María Luisa Ojeda, Fátima Nogales, José A. Carrasco López, María del Carmen Gallego-López, Olimpia Carreras, Ana Alcudia, Eloísa Pajuelo

**Affiliations:** 1 Departamento de Fisiología, Facultad de Farmacia, Universidad de Sevilla, 41012 Sevilla, Spain; ojedamuri11@us.es (M.L.O.); mgallego3@us.es (M.d.C.G.-L.); olimpia@us.es (O.C.); 2 Departamento de Microbiología y Parasitología, Facultad de Farmacia, Universidad de Sevilla, 41012 Sevilla, Spain; epajuelo@us.es; 3 Departamento de Química Orgánica y Farmacéutica, Facultad de Farmacia, Universidad de Sevilla, 41012 Sevilla, Spain; aalcudia@us.es

**Keywords:** selenite, nanoparticles, microbiota, total bile salts, GLP-1

## Abstract

Adolescence is a period during which body composition changes deeply. Selenium (Se) is an excellent antioxidant trace element related to cell growth and endocrine function. In adolescent rats, low Se supplementation affects adipocyte development differently depending on its form of administration (selenite or Se nanoparticles (SeNPs). Despite this effect being related to oxidative, insulin-signaling and autophagy processes, the whole mechanism is not elucidated. The microbiota–liver–bile salts secretion axis is related to lipid homeostasis and adipose tissue development. Therefore, the colonic microbiota and total bile salts homeostasis were explored in four experimental groups of male adolescent rats: control, low-sodium selenite supplementation, low SeNP supplementation and moderate SeNPs supplementation. SeNPs were obtained by reducing Se tetrachloride in the presence of ascorbic acid. Supplementation was received orally through water intake; low-Se rats received twice more Se than control animals and moderate-Se rats tenfold more. Supplementation with low doses of Se clearly affected anaerobic colonic microbiota profile and bile salts homeostasis. However, these effects were different depending on the Se administration form. Selenite supplementation primarily affected liver by decreasing farnesoid X receptor hepatic function, leading to the accumulation of hepatic bile salts together to increase in the ratio Firmicutes/Bacteroidetes and glucagon-like peptide-1 (GLP-1) secretion. In contrast, low SeNP levels mainly affected microbiota, moving them towards a more prominent Gram-negative profile in which the relative abundance of *Akkermansia* and *Muribaculaceae* was clearly enhanced and the Firmicutes/Bacteroidetes ratio decreased. This bacterial profile is directly related to lower adipose tissue mass. Moreover, low SeNP administration did not modify bile salts pool in serum circulation. In addition, specific gut microbiota was regulated upon administration of low levels of Se in the forms of selenite or SeNPs, which are properly discussed. On its side, moderate-SeNPs administration led to great dysbiosis and enhanced the abundance of pathogenic bacteria, being considered toxic. These results strongly correlate with the deep change in adipose mass previously found in these animals, indicating that the microbiota–liver–bile salts axis is also mechanistically involved in these changes.

## 1. Introduction

Adolescence is a period of intense endocrine changes, resulting in the modulation of body weight and composition, where obesity and anorexia processes are lately increasing [1,2]. Selenium (Se) is a trace element with excellent antioxidant and anti-inflammatory properties mediated by different selenoproteins such as glutathione peroxidase (GPx) [3]. Correct oxidative balance is deeply related to the endocrine signaling process and cell proliferation and differentiation [4]. It has been recently found that Se is deeply related to white adipose tissue (WAT) metabolism during adolescence [5]. However, its effects clearly differ depending on the form of administration. Therefore, supplementation with inorganic selenite at low doses contributed to the increase in WAT adipogenesis via the insulin signaling pathway and by modulating adipokine secretion, especially by decreasing lipocalin-2 (LCN2). While the administration of low doses of Se nanoparticles (SeNPs), which have a high surface area and solubility, prevented fat deposits in WAT. This treatment decreased insulin signaling and increased forkhead box O3a (FOXO3a), affecting autophagy and lowering inflammation. However, since both forms of Se administration increased the activity of the antioxidant enzyme GPx1 in the same way, it is inferred that their contrasting effects on WAT adipocytes are GPx1-independent [5]. Therefore, there must be additional factors that contribute to these deep changes in lipid homeostasis.

It is well known that Se plays an important role in the maintenance of gastrointestinal tract (GIT) health by enhancing its antioxidant function, mainly by increasing GPx2 activity. It contributes to the reduction in the production of reactive oxygen species to a large extent, reducing the damage to the intestinal mucosa. In addition, Se also reduces GIT damage modulating autophagy and apoptosis pathways [6]. GIT oxidative balance exerts an important impact on the structure and function of GIT microbial communities [7]. Therefore, Se clearly modulates gut microbiota contributing to balancing the microbial flora [8]; however, this modulation also depends on Se dose and administration form [9,10]. The gut microbiota has been recognized as a significant environmental factor in the maintenance of energy homeostasis and host immunity. For instance, it is well established that obesity, and therefore, WAT mass development, is related to a higher Firmicutes to Bacteroidetes (F/B) ratio [11]. Some studies clearly found that Verrucomicrobia were negatively associated with obesity and lipid homeostasis; specifically, *Akkermansia municiphila* is present at a lower abundance in overweight/obese children and adults as compared to healthy people [12,13]. In this context, a growing body of evidence suggests that the gut microbiota regulates host metabolism through close crosstalk with liver and adipose tissues. It modulates energy expenditure and WAT deposits by producing specific metabolites such as short-chain fatty acids (SCFA) which modulate insulin secretion, lipopolysaccharide (LPS) and peptidoglycans. However, when dysbiosis appears these elements may act as central factors in the pathogenesis of inflammation, insulin resistance and obesity [14].

The communication between the gut and these tissues, namely the liver and the adipose tissue, works bidirectionally [15]. Therefore, the liver and the adipose tissue also communicate with the intestine and the gut-microbiota axis by secreting different organokines. Specifically, the liver communicates with the gut through the biliary system and with the systemic circulation by releasing bile acids (BAs) and different hepatokines such as the fibroblast growth factor 21 (FGF21), a pleotropic organokine with beneficial effects on maintaining energy homeostasis [16]. Recently several authors have pointed to FGF21 as a negative regulator of hepatic BAs metabolism [17,18]. Primary BAs are synthesized in liver mainly by the enzyme cholesterol 7-alpha-hydroxylase (CYP7A1) from cholesterol, latter conjugated BAs are secreted to duodenum in the form of bile salts through the bile salt export pump (BSEP). BAs are mainly known for their intestinal function, being necessary for fat digestion and absorption and, therefore, for lipid and energy homeostasis; however, currently, they are also considered important systemic signaling metabolites involved in body weight [19,20,21]. In the terminal ileum, the majority of BAs are actively reabsorbed through apical sodium-dependent bile salt transporter (ASBT) and return to the liver via the portal vein to inhibit BAs synthesis. The remaining 5–10% are transformed into secondary BAs by the microbiota in the colon and are passively reabsorbed or excreted. The transformation from primary to secondary BAs (deconjugation and dihydroxylation) is facilitated by bile salt hydrolases (BSH) and 7α-dehydroxylase enzymes expressed by the gut microbiome, including the genera *Bacteroides*, *Clostridium*, *Eubacterium*, *Lactobacillus* and *Escherichia*; most of them are anaerobes [19,22,23,24]. Thus, the microbiota plays an important role in BAs homeostasis.

Two mechanisms have been suggested to inhibit CYP7A1 in the liver and thus primary BAs synthesis and in both cases, farnesoid X receptor (FXR) is involved. Firstly, hepatic FXR expression inhibits CYP7A1 and upregulates BSEP contributing to eliminating the accumulation of BAs in the liver. Secondly, in the intestine, FXR induces fibroblast growth factor FGF15/19, which inhibits hepatic CYP7A1 after reaching the liver [25,26]. In this last mechanism both colon microbiota and secondary BAs are involved, since decreasing in microbiota BSH and 7α-dehydroxylase activities increases tauro-α-muricholic acid levels, which antagonizes intestinal FXR activity and reduces FGF15/19, stimulating primary BAs synthesis in hepatocytes. Thus, the gut-to-liver axis plays a critical role in regulation of primary and secondary BAs homeostasis, which also contributes to lipid and glucose balance [15,27,28]. On the other hand, BAs also have direct and indirect influence on the composition of the gut microbiome through the ability to harm bacterial cell membranes and through the promotion of antimicrobial peptides [29].

Previous studies have described that chronic inorganic Se supplementation clearly increases primary BAs biosynthesis in the liver, decreasing BA metabolites [30]; however, there is no information relative to BAs cycle, such as BAs concentration in bile, serum or porta-hepatic vein. Taking into account that colon microbiota could be playing an important role in this homeostasis, and therefore, in lipid utilization and WAT mass growth, the aim of this study was to explore the effects of supplementation with low doses of either selenite or SeNPs during adolescence on the colon microbiota and on BAs homeostasis; since this could be another important mechanistic-axis by which Se supplementation could be contributing to modulate WAT mass homeostasis, apart from its well-known antioxidant action.

## 2. Materials and Methods

### 2.1. Animals

All animal-care procedures and experimental protocols were in accordance with the guidelines of the European Union Council (Directive 2010/63/UE) and Spanish Royal Decree (BOE 34/11370, 2013) concerning the protection of experimental animals. Approval for the research protocol was granted by both the Ethics Committee of the University of Seville (CEEA-US2019-4) and the Junta de Andalucía (05-04-2019-065).

In this experimental design, a total of 24 adolescent male Wistar rats from the Centre of Production and Animal Experimentation at the Vice-Rector’s Office for Scientific Research, University of Seville were utilized. They were received on the postnatal day (PND) 21. After one week of acclimation to housing conditions in groups of two rats per cage, the experimental protocol was conducted after 3 weeks from PND 28 to PND 47. This period corresponds to adolescence in Wistar rats [31]. The animals were kept at an automatically controlled temperature (22–23 °C) and in a 12 h light–dark cycle (09:00 to 21:00) by providing environmental enrichment.

At PND 28, rats were assigned randomly to one of the four experimental groups (*n* = 6/group) based on their respective treatments: the control group (C), which received normal drinking water, the low-sodium selenite supplementation group (S), which was given drinking water supplemented with low Na_2_SeO_3_; the low-SeNP-supplementation group (NS), which were exposed to low SeNP supplementation in drinking water; and the moderate SeNP supplementation group (NSS), which were given a moderate SeNP supplementation in drinking water. Drinking water supplemented and administered ad libitum in the four groups.

All experimental groups had access to the standard pellet diet (LASQCdiet^®^ Rod14-R; Märkische, Germany) containing 0.2 ppm of Se in the form of Na_2_SeO_3_, which was available ad libitum. S and NS rats were given an additional 0.14 ppm of Se in the form of anhydrous sodium selenite (Panreac, Barcelona, Spain) and SeNPs in their drinking water throughout the experimental periods. NSS rats received 1.4 ppm of extra Se solely in the form of SeNPs in their drinking water. With this low Se supplementation, the rats consumed in average 6 µg/day of Se, which was equivalent to 500 μg/kg of dietary Se, consistent with the highest GPx activity in rat plasma and liver [32]. Moderate-SeNPs supplementation (1.4 ppm Se) was tenfold higher in order to investigate its potential toxic effect.

### 2.2. SeNPs Development

SeNPs were synthesized at the Department of Organic and Medicinal Chemistry, Faculty of Pharmacy, University of Seville, Spain, with the following chemicals: Se tetrachloride (SeCl_4_), ascorbic acid (C_6_H_8_O_6_) and poly(sodium 4-styrenesulfonate) (PSSS) (Sigma-Aldrich, Madrid, Spain). The SeNPs were freshly prepared according to the previously described procedure [5,33], which involved the use of optimal amounts of ascorbic acid as a chemical reductant to minimize the use of more toxic alternatives such as sodium borohydride. The precipitation of SeNPs was carried out to obtain the smaller-sized nanoparticles (less than 50 nm).

### 2.3. Nutritional Control

During the whole experimental protocol, body weight, liquid and solid intakes were monitored daily using an analytical balance. The amount of food and water ingested was calculated by measuring the difference between the weight of these parameters every morning and the next day. To calculate the Se intake, the concentration of Se (ppm) in the diet and drinking water was determined, and this value was multiplied by the amount of food and water ingested by the rats every day. These measurements were always taken at 9:00 a.m.

### 2.4. Samples and Anthropometric Measurements

After the experimental period ended, the rats were placed in individual metabolic cages and fasted for 12 h. Subsequently, the adolescent rats were anesthetized with an i.p. injection of 28% *w*/*v* urethane at a dose of 0.5 mL per 100 g of body weight. At the same time, the cranium–caudal length was measured, and the body mass index (BMI) were calculated using the formula: body weight (g)/length^2^ (cm^2^). Circulating blood was obtained via heart puncture, and porta-hepatic blood after opening the abdominal cavity and inserting into the portal vein a non-obstructive plastic catheter to draw portal blood. In both cases, the blood was collected in tubes without anticoagulants, which were incubated at room temperature for 30 min and then centrifuged at 1300× *g* for 15 min to prepare the serum.

Immediately after opening the abdomen, liver and duodenum were removed and rinsed with ice-cold saline to be weighed and frozen in liquid nitrogen, to finally be stored at −80 °C. Before freezing, duodenal mucosa was obtained by scraping the duodenum with two slides and then weighed. To calculate the total and serosa area the duodenal perimeter was measured [34]. Simultaneously, cecum feces were collected in sterile tubes and quickly frozen in liquid nitrogen and stored at −80 °C for subsequent analysis of the bacterial composition.

### 2.5. DNA Extraction and High-Throughput Sequencing

The total genomic DNA from cecum feces (0.1 g) stored at −80 °C was extracted using the FavorPrep Stool DNA Isolation Mini Kit (Favorgen Biotech Corp., Vienna, Austria), following the manufacturer’s instructions. DNA integrity was analyzed by gel electrophoresis in a 1.5% agarose gel upon visualization of clear gel with defined DNA bands or slight smear. When needed, purification was performed with magnetic beads Sera-Mag Select (Cytiva, Cornellá de Llobregat, España). The final concentration (minimum of 5 ng/µL) was determined using a Qubit 2.0 fluorometer and Qubit dsDNA BR kit. The concentration of DNA in our samples ranged from 14.2 to 34.4 ng/µL. Quality control for integrity post-library preparation was carried out by electrophoresis in a 1.5% agarose gel in TAE buffer. The observation of clear gel with a defined band of expected size was indicative of good DNA integrity. The concentration (minimum of 4 nM) was determined using Qubit 2.0 fluorometer and Qubit dsDNA BR kit. The concentration of DNA in our particular library samples ranged from 34.4 to 60.4 ng/µL.

High-throughput sequencing of fecal microorganisms was performed through the company StabVida (Caparica, Portugal). The V3-V4 hypervariable region of the 16S rRNA was paired-end sequenced (2 × 300 bp reads) using the primers F (341F): 5′CCTACGGGNGGCWGCAG3′ and R (785R): 5′ GACTACHVGGGTATCTAATCC 3′ and the Illumina MiSeq platform with MiSeq Reagent Kit v3 [35]. The raw Illumina sequences were demultiplexed and quality checked using the FastQC software, applying 25% output deficit tolerance in the number of reads and more than 60% of the bases higher than Q30 at 2 × 300 bp. Low-quality reads were removed. The analysis of the generated raw sequence data was carried out using QIIME2 v2022.2 [36]. The reads were denoised by trimming and truncating low-quality regions, dereplicating the reads and filtering chimeras in order to reconstruct amplicon sequence variants (ASVs). Denoising was performed using the Divisive Amplicon Denoising Algorithm plugin (DADA 2; [37]). Rarefaction curves were completed in order to evaluate the alpha diversity of the samples and assure that the depth of sequencing was sufficient enough to represent their identities. Rarefaction curves were generated for categories and individual samples by randomly selecting different numbers of sequences and analyzing the operational taxonomic units (OTUs) detected at each fraction.

The reads were organized in OTUs and then classified by taxon using a fitted classifier. The scikit-learn classifier was used to train the classifier using the SILVA (release 138 QIIME) database, with a clustering threshold of 99% similarity. For classification purposes, only OTUs containing at least 10 sequence reads were considered significant.

### 2.6. Systemic Signalers

Luminex xMAP technology-based Milliplex^®^ MAP Rat Panel (Merck Millipore, Darmstadt, Germany) was used to measure serum signalers such as the hepatokin FGF21, the epidermal growth factor (EGF) and the incretin glucagon-like peptide-1 (GLP-1). The preparation of samples, reagents and standards was carried out following the manufacturer’s instructions. The standard curve pattern was performed by successive dilutions of the stock concentration for each biomarker. Equipment conditions were set following the manufacturer’s instructions using the reader LABScan 100analyzer (Luminex Corp., Austin, TX, USA).

### 2.7. Total Bile Acid Homeostasis

Total bile acids (TBA) were determined in liver homogenates and serum from systemic blood and porta vein, by ELISA, using the “Rat Total Bile Acid ELISA Kit” from MyBioSource, Inc. (San Diego, CA, USA). Liver samples were homogenized (1:10 *w*/*v*) in ice-cold PBS using a Potter homogenizer (Pobel 245432, Madrid, Spain). The homogenates were incubated at 4 °C for 20 min and then, they were centrifuged at 12,000× *g* for 20 min. This ELISA kit was carried out with the reagents supplied by the manufacturer and according to their specifications, in a 96-well plate. The TBA standards and the samples are added to a TBA monoclonal antibody precoated microplate. The TBA concentration (ng/mL) in the samples was quantified by comparing its absorbance at 450 nm with that of the standard curve.

### 2.8. Serum LPS Levels

The quantification of serum LPS was performed using the “Rat Lipopolysaccharides ELISA Kit” from Cusabio (Houston, TX, USA) and following the instructions of the commercial kits. It is a Sandwich ELISA kit that contained a 96-well plate precoated with LPS-specific antibody and with a sensitivity of 0.039 ng/mL. LPS levels (ng/mL) in the samples are quantified by comparing their absorbance at 450 nm with that of the standard curve.

### 2.9. Immunoblotting Assays FXR

FXR expression in liver homogenates was determined with the protein immunodetection technique. Each liver sample contained 100 µg of protein, previously determined by the method by Lowry et al. [38]. Proteins were separated on a polyacrylamide gel (9%) and transferred to a nitrocellulose membrane (Immobilon-P Transfer Membrane; Merck Millipore) using a blot system (Transblot; BioRad, Madrid, Spain).

FXR mouse monoclonal IgG (Santa Cruz Biotechnology, Heidelberg, Germany) (1:500); and monoclonal mouse anti-β-actin IgG1A5441 (Sigma-Aldrich) (1:10,000), as a load control were incubated overnight at 4 °C. The antigen–antibody complex was detected by incubation of the membranes with the secondary antibody: goat Anti-Mouse IgG (H + L)-HRP Conjugate (BioRad) and revealed using Luminol ECL reagent (GE Healthcare and Lumigen Inc., Buckinghamshire, UK). The bands of interest were quantified by using densitometry with the ImageJ program. The results were expressed as a percent of arbitrary relative units, referring to values in control animals, which were defined as 100%.

### 2.10. Statistical Analysis

The study data were analyzed using statistical software (GraphPad InStat 3; San Diego, CA, USA) with one-way ANOVA, and the results were presented as means ± standard errors of the mean (SEMs), based on 6 animals in each group. Statistical significance was set at *p* < 0.05. If differences were detected with ANOVA with *p*-values < 0.05, significant differences between means were further evaluated using the Tukey–Kramer test.

## 3. Results

### 3.1. Duodenal Parameters: Sodium Selenite Supplementation Increases EGF Serum Level, and Low-SeNP Therapy Increases Mucosa Layer Weight and Duodenum Perimeter

Table 1 shows that neither of the two forms of low Se supplementation to adolescent rats affected their body weight, nor solid or liquid intake. These animals received twice the amount of Se than control rats daily. However, NSS rats presented a clear decrease in solid and liquid intake with regard to the rest of the groups, presenting the lowest body weight. NSS rats received daily 10 times more Se than C rats. NSS rats also presented significantly lower duodenum weight but higher mucosa weight than C and S rats. NS animals displayed higher duodenum mucosa weight than S rats. Both SeNP-supplemented groups (NS and NSS) had greater duodenal perimeter than C group. Finally, serum EGF concentration was significantly increased in the S group compared to the rest of the groups. NS and NSS rats had lower EGF values than control ones, being these values greater in NS than in NSS animals.

### 3.2. Low SeNP Supplementation Increases the Main Phyla Related to Weight Loss and Lower BMI, While Sodium Selenite Therapy Increases Those Related to Butyrate Generation and GLP-1 Production

**Figure 1:**
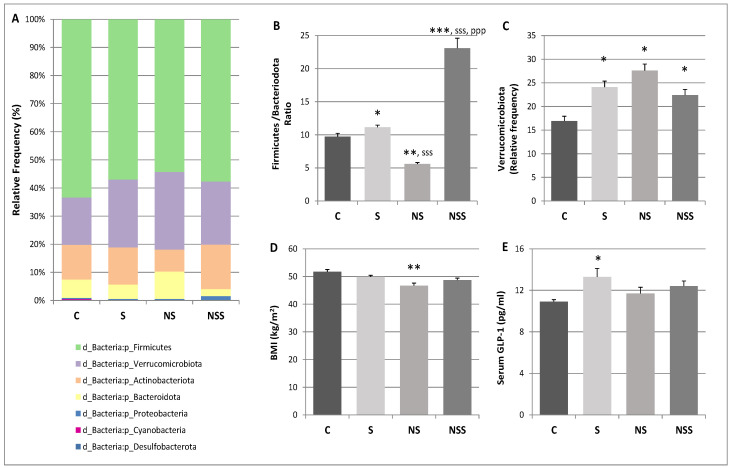
(**A**). Mean relative abundance of main Phyla, (**B**). Firmicutes/Bacteroidota ratio, (**C**). Relative Verrucomicrobiota abundance, (**D**). Body Max Index (BMI) and (**E**). Serum GLP-1 values. The mean ± SEM values of the results were analysed using a multifactorial one-way ANOVA and Tukey’s test. Groups label: C (control), S (low-sodium selenite), NS (low SeNPs) and NSS (moderate-SeNPs). Significance: vs. C, * *p* < 0.05, ** *p* < 0.01, *** *p* < 0.001; vs. S, ^sss^ *p* < 0.001; vs. NP, ^ppp^ *p* < 0.001.

A taxonomical classification was performed using the SILVA database (release 138 QIIME) trained using scikit-learn. Figure 1A shows the relative abundance of main taxons at the level of Phylum. The results showed that four main phyla accounted for more than 80% of the bacterial populations, namely, Verrucomicrobiota, Firmicutes, Actinobacteriota and Bacteroidota. Much less represented were Proteobacteria, Cyanobacteria and Desulfobacterota. The F/B ratio was also calculated (Figure 1B), compared to control rats, this ratio was significantly increased in S rats and decreased in NS ones. Moreover, this ratio was significantly increased in moderate-SeNPs animals vs. the rest of the groups (*p* < 0.001). Concerning Verrucomicrobiota (Figure 1C), the relative abundance of this phylum was significantly increased in all the Se treatments, but particularly in NS. Final BMI was represented in Figure 1D, being significantly decreased in NS rats with respect to C ones. Figure 1E shows serum levels of the incretin GLP-1, these levels were significantly increased in the S group vs. C rats.

### 3.3. Low SeNP Supplementation Enhances the Relative Abundance of Gram-Negative Bacteria

The relative abundance of the most represented phyla of Gram-positive bacteria (namely, the sum of Firmicutes + Actinobacteriota) and Gram-negative bacteria (namely, the sum of Verrucomicrobiota + Bacteroidota + Proteobacteria) was calculated (Table 2). The overall abundance of Gram-negative bacteria showed a tendency to increase in the three Se-treated groups. However, this increase was only significant in NS group vs. C (*p* < 0.001). This upgrowth of Gram-negative strains may be the reason for the significative accumulation of LPS in serum in this group (*p* < 0.05). The main phyla accounting for the increase in Gram-negative population were Verrucomicrobiota + Bacteroidota. NS rats also presented a significant decrease in the Gram-positive bacteria population (*p* < 0.05) compared to the C group.

### 3.4. Taxonomical Classification at Level of Genus

**Figure 2:**
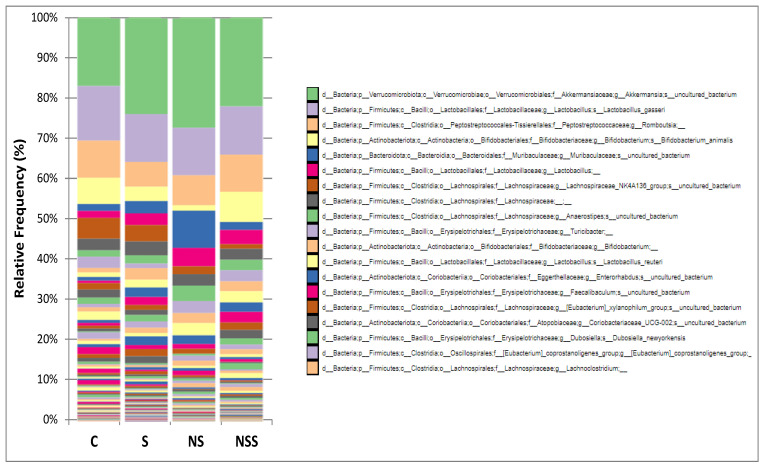
Mean relative abundance of main Genera by treatment. Groups: C, control; S, low-sodium selenite; NS, low SeNPs; NSS, moderate-SeNPs.

Figure 2 shows the relative abundance of the first 19 main genus by order of appearance according to their abundance in each experimental group. Being the first genus the more abundant. These results were evaluated in Table 3, Table 4 and Table 5.

### 3.5. Bacteria Belonging to Clostridium Clusters IV, XI and XIV Were Decreased after Sodium Selenite Exposure

**Figure 3:**
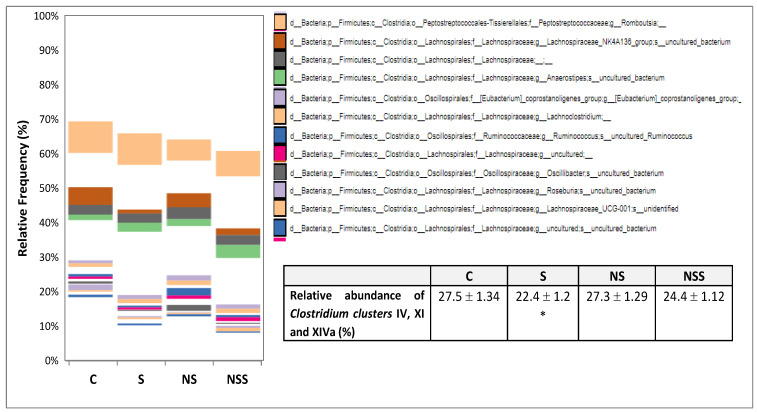
Mean relative abundance in *Clostridium* clusters IV, XI and XIVa, all bacterial involved in bile acid 7α-dehydroxylation. The mean ± SEM values of the results were analysed using a multifactorial one-way ANOVA and Tukey’s test. Groups label: C (control), S (low-sodium selenite), NS (low SeNPs) and NSS (moderate-SeNPs). Significance: vs. C, * *p* < 0.05.

The first 12 bacteria belonging to *Clostridium* clusters IV, XI and XIVa were shown in Figure 3, being the first genus and the most abundant one. Their accumulative relative abundance was determined by the group of treatment, being significantlt decreased in the S group vs. C rats (*p* < 0.05).

### 3.6. Total Bile Salts (TBS) Homeostasis: Sodium Selenite Supplementation Decreases FXR Expression, Leading to the Accumulation of TBS in the Liver, Low-SeNP Treatment Reduces the Enterohepatic Circulation of TBS, Resulting in Lower Lipid Absorption in the Intestine

**Figure 4:**
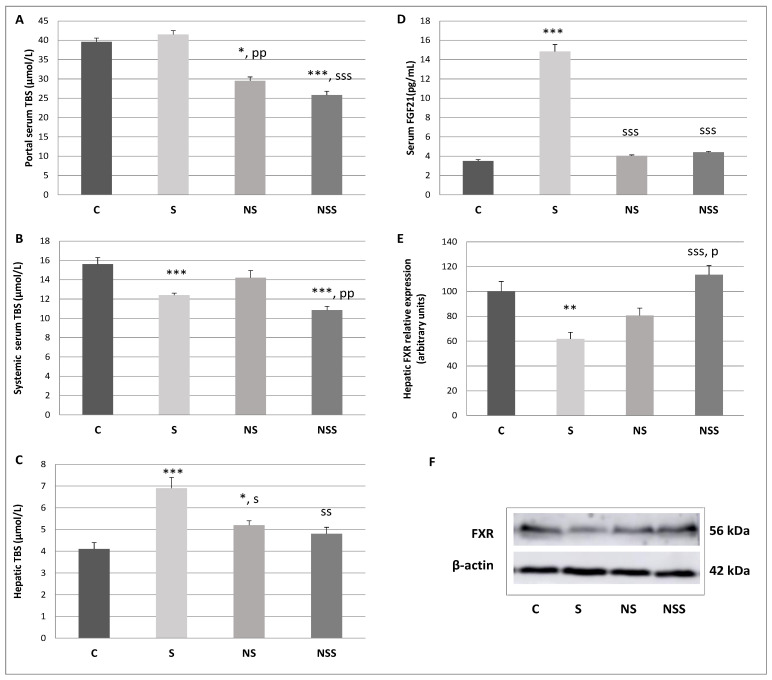
Total bile salt (TBS) homeostasis. (**A**). Portal serum TBS concentration, (**B**). Systemic serum TBS concentration, (**C**). Hepatic TBS concentration, (**D**). serum FGF21 concentration, (**E**). FXR hepatic relative expression, (**F**). Representative Western blots of proteins (normalized to β-actin). The mean ± SEM values of the results were analysed using a multifactorial one-way ANOVA and Tukey’s test. Groups label: C (control), S (low-sodium selenite), NS (low SeNPs) and NSS (moderate-SeNPs). Significance: vs. C, * *p* < 0.05, ** *p* < 0.01, *** *p* < 0.001; vs. S, ^s^ *p* < 0.05, ^ss^ *p* < 0.01, ^sss^ *p* < 0.001; vs. NP, ^pp^ *p* < 0.01, ^p^ *p* < 0.05.

S adolescent rats presented significantly lower levels of systemic TBS than C rats; and higher TBS hepatic and serum FGF21 levels, together to lower hepatic FXR expression than the rest of the groups (Figure 4). On its side, NS rats showed higher TBS hepatic levels than C rats but lower TBS enterohepatic serum levels than C and S groups. Finally, NSS animals presented lower TBS portal serum levels than C and S groups, and lower TBS systemic serum levels than C and NS groups.

### 3.7. Contrasting Effects of Low Se Supplementation in the Colonic OTUs of Adolescent Rats in Relation to TBS and Lipid Homeostasis and Weight Management

Table 3 shows a summary of OTUs whose relative abundance increased in both S and NS groups compared to C rats; 13 OTUs showed increased representation after Se treatment. By contrast, Table 4 describes a summary of OTUs whose relative abundance decreased in both S and NS groups compared to C rats; in this case, 11 OTUS displayed lower relative abundance than the control and 1 was undetected after Se treatment. Finally, Table 5 presents a summary of OTUs whose relative abundance was contrasting in S and NS groups; 11 OTUS showed upgrowth after selenite treatment, and only 1 showed diminished abundance. After SeNPs treatment only 1 OTUs increased its relative abundance, whereas 7 decreased their representation and 4 were not detected. In all three tables, the genus appeared in order of their abundance in control rats. Since we have identified five main processes or aspects related to the bacterial main function, they have been assigned to five main roles: (a) weight management and lipid storage; (b) butyrate production; (c) bile salt hydrolase activity; (d) bile acid 7α-dehydroxylation activity; and (e) those linked to dysbiosis and inflammation.

## 4. Discussion

### 4.1. Duodenal Parameters: Sodium Selenite Supplementation Increases EGF Serum Level and Low-SeNP Therapy Increases Mucosa Layer Weight and Perimeter

Low Se supplementation in adolescent rats for 3 weeks, independently of the form of administration, did not affect solid or liquid intake, or final body weight. However, lipid homeostasis was altered after these treatments, since the same results of significantly lower fat deposits after SeNPs administration and higher adipocyte sizes with hyperinsulinemia in selenite-supplemented adolescent rats were found [5]. In this context, even though sodium selenite supplementation did not affect duodenum macroscopically, it promoted the secretion of the growth factor EGF in the serum. This factor enhances cellular proliferation and differentiation; in addition, it also functions as a GIT mucosal protective factor improving nutrient absorption [61,62]. In fact, serum EGF levels have been correlated with different patterns of bowel inflammation, epithelial development and wounds in duodenum, which are a reflection of EGF intestinal status [63]. Moreover, in vitro models have demonstrated a central role for EGF receptors in the trans-differentiation of pancreatic acinar and ductal cells in endocrine islet cells, which is an essential point in beta-cell mass regulation and insulin secretion [64]. Therefore, selenite promotes duodenum function and nutrient absorption, by enhancing EGF secretion, which also contributes to secreting insulin, a key factor to promote the largest adipocyte and an adipokine secretion pattern related to insulin sensitivity and general growth [5].

In contrast, low SeNP administration led to lower secretion of EGF. This fact could contribute to the hipoinsulinemia previously reported in these animals [5]. However, these rats presented a higher duodenum perimeter than control rats and a higher duodenum mucosa weight than S rats. These results are according to those found in [10], in which duodenum mucosal of broilers after low SeNPs administration present a greater density of mucosal layer in duodenum without histopathological damage. These authors indicate that SeNPs are non-toxic, while they exhibit a higher absorption in intestines and a lower retention in tissues involved in detoxification, such as the liver, compared to selenite. In fact, recently, it is suggested that the dietary nano-Se intake promotes the recovery of antioxidant enzyme activities, mainly by increasing Catalase expression in GIT, to a great extent than other Se compounds. This fact enhances protein repair, alleviates inflammatory response and increases GIT length [6,65].

Finally, moderate-SeNP-supplemented rats presented extremely low levels of serum EGF, lower duodenum weight and, probably to improve nutrient absorption, greater duodenum perimeter and mucosal weight than control rats. These facts, together with lower solid and liquid intake, could indicate a toxic effect of this treatment which collaborates to malnutrition.

### 4.2. Low SeNP Supplementation Increases the Main Phyla Related to Weight Loss and Lower BMI, While Sodium Selenite Therapy Increases Those Related to Butyrate Generation and GLP-1 Production

In this study, Se administration during adolescence clearly affected the relative abundance of the main taxons at the level of Phylum. The gut–microbiota interaction has been pointed to as a hot topic of research in the treatment of obesity and related metabolic diseases by influencing energy metabolism and the immune system. Therefore, these changes at Phylum level could give information relative to energy used and adipose tissue homeostasis. There is a wide corpus of publications which suggest correlations between BMI and the presence of specific gut microorganisms are considered microbial biomarkers that are linked to obesity and high WAT mass [66]. For instance, it is well established that obesity is related to a higher F/B ratio [11]. In contrast, low F/B ratio appeared in lean rats and in rats exposed to antioxidant diets, being consistent with a healthy GIT [7]. Recently, previous studies found that Verrucomicrobia was negatively associated with obesity and lipid homeostasis in humans [12,13]. The only known representant of this phylum in the human gut microbiota is the anaerobic *Akkermansia municiphila*. This microorganism was reported to be present in a lower abundance in overweight/obese children and adults compared to healthy people. This is considered a pivotal species in the intestinal mucous layer due to its capacity to degrade mucin and produce acetate and propionate. Moreover, the low abundance of this microorganism is related to increased intestinal permeability and inflammation [13]. Currently *A. muciniphila* is consistently correlated with obesity and it is used as a therapeutic supplement in humans [40]. Interestingly, it is also considered a helpful strategy to reduce the production of oxidative stress along the GIT [67].

According to that, low selenite supplementation in adolescent rats affected the relative abundance of the main Phyla. Specifically, it favored the relative abundance of Verrucomicrobiota and Bacteroidota and decreased that of Firmicutes and Actinobacteriota. In these rats, the ratio F/B was slightly increased. However, Verrucomicrobiota was also upregulated, as this last phylum was clearly inversely related to obesity and WAT development [39]. Therefore, S adolescent rats, despite presenting higher insulin sensitivity in WAT and larger adipocyte size [5], had normal body weight and BMI at the end of the experimentation process. In contrast, low-SeNP-treated rats presented a lower F/B ratio and higher Verrucomicrobiota abundance than the control and low-selenite-treated rats. These results clearly coincide with a lower BMI at the end of the experimental procedure and a decrease in WAT somatic index [5], but also a higher antioxidant activity on GIT. Therefore, our results indicate that the low-Se-supplementation therapies used in this study affect final WAT development and BMI partially by modulating colon microbiota.

SCFAs produced through fiber fermentation in the colonic region are known to regulate the proper function, motility and integrity of the GIT, contributing to modulating glucose and lipid metabolism in a beneficial way. However, excessive SCFAs might increase intestinal energy harvesting capabilities and induce obesity [49]. SCFAs also improve glucose homeostasis and strengthen satiety by increasing the production of the incretin GLP-1 in the intestine [68]. Therefore, GLP-1 protects against hyperglycaemia by enhancing insulin secretion and inhibiting glucagon secretion. Within the gut, it also acts to inhibit gastric motility and secretion, and in the brain, it engages a range of neural circuits to regulate appetite and reward-related behaviours [69]. Butyrate is the main SCFA related to GLP-1 induction [70]. In this context, a higher F/B ratio is related to greater delivery of butyrate vs. acetate and propionate [71]. In fact, S rats presented a higher F/B ratio that correlated to higher GLP-1 serum values, which is in accordance with the hyperinsulinemia previously described in these adolescent rats [5]. Once more, in contrast, NS rats showed a lower F/B ratio, and the GLP-1 serum levels were not increased, according to the lower insulin serum levels that these animals reported in previous papers [5]. However, additional mechanisms are necessary to be explored to explain the serum insulin results found after both treatments. On the other hand, the impressive increase in the ratio of F/B in rats treated with excess SeNPs, together with the increase in Enterobacteriaceae (potentially pathogenic), revealed the high dysbiosis provoked by toxicity.

### 4.3. Low SeNP Supplementation Enhances the Relative Abundance of Gram-Negative Bacteria

Relative to the percentage of Gram-positive and Gram-negative bacterial populations in the colon, all Se-treated groups displayed a tendency towards higher relative expression of Gram-negative species. This tendency was mainly due to an increase in the phylum Verrucomicrobiota, especially related to *Akkermansia muciniphila* (Figure 1 and Figure 2) which, as was previously mentioned, is clearly with weight loss [72]. Moreover, low SeNP supplementation also led to an important increase in the phylum of Gram-negative Bacteroidota, mainly by increasing the abundance of the beneficial bacterial related to longevity Muribaculaceae, which significantly contributes to increasing the Gram-negative population (Figure 2) [73]. Therefore low-SeNP-treated rats presented higher serum LPS levels than control animals. Similar results with SeNPs therapies in mice have been described, since SeNPs treatment reversed perturbed gut microbiota by decreasing the F/B ratio, and increasing the abundance of beneficial bacteria such as *Akkermansia, Muribaculaceae, Bacteroides and Parabacteroides* [74]. It is also important to point out for future discussion aspects, that low levels of bile salts favor the proliferation of Gram-negative bacteria, while high levels of bile salts favor the proliferation of Gram-positive bacteria and the reduction in the Gram-negative *Bacteroides* [43,75]. NSS adolescent rats showed a slight tendency to increase the Gram-negative population, with 10% of them belonging to the phylum Proteobacteria. Proteobacteria includes a wide variety of pathogenic genera, such as *Escherichia*, *Salmonella*, *Vibrio*, *Helicobacter*, *Yersinia* and *Legionella*, to name a few [76]. Therefore, NSS treatment seems to have potential toxic effects.

Overall, these results on duodenal development and on colonic main phyla modulation, indicate that low SeNP supplementation during adolescence is an effective therapy to decrease BMI and lipid accumulation, with a promising application in obesity during adolescence. In contrast, sodium selenite treatment could be useful during catabolic process, such as anorexia, by inducing the insulin-signaling process since it clearly increases GLP-1 serum levels.

### 4.4. TBS Homeostasis: Sodium Selenite Supplementation Decreases FXR Expression, Leading to the Accumulation of TBS in the Liver, Low-SeNP Treatment Reduces the Enterohepatic Circulation of TBS, Resulting in Lower Lipid Absorption in the Intestine

Sodium selenite supplementation during adolescence not only alters colon microbiota population and BMI, but it also affects TBS homeostasis and the Se supplementation incidence in a different way. The main functions of BAs include emulsifying and digesting fat to be absorbed, regulating and excreting cholesterol and exerting antimicrobial effects, apart from a novel general endocrine function related to lipid and glucose metabolism [77]. Several anaerobes are essential in the regulation of the BAs pool, the deconjugation by microbial BSH, the 7α-dehydroxylation activity which converts the primary BAs into secondary (the most important and the most physiologically significant conversion of BAs), and the esterification of BAs by the intestinal microbiota, can dramatically change their physicochemical properties and subsequently affect lipid intestinal absorption [77]. Most colon microorganisms are anaerobes which play a major role in the homeostasis of BAs, being tolerant to BAs. Those with 7α-dehydroxylation activities are particularly outstanding, such as a small number of bacterial species belonging to the *Clostridium* clusters IV, XI and XIVa. In contrast, a high colonic BAs concentration leads to a reduction in Gram-negative *Bacteroides* [43]. According to that, BAs homeostasis selectively restrains certain microbial species and subsequently affects the composition of the colon microbiota, and vice versa [77]. However, the complex interactions between BAs and host-microbiome in the gut–liver axis, which also compromises lipid metabolism and WAT storage, are only beginning to be understood [75].

Previous studies have described that chronic Se supplementation in the form of selenate by different mechanisms alters hepatic energy and fatty acid metabolism in mice, contributing to an increase in weight and WAT mass. Among this mechanism, it is well-described that Se clearly increases primary BAs biosynthesis in the liver [30]. Moreover, these authors suggest that the Se-impaired peroxisomal oxidation and the biosynthesis of BAs may lead to a change in glucose homeostasis because BAs regulate glucose metabolism and increase insulin sensitivity through intestinal FXR and TGR5. These effects do not seem to be related to its antioxidant action via the selenoprotein GPx, but to a downstream energy metabolism dependent on acetyl-CoA. In agreement with this hypothesis, in our work, selenite supplementation in adolescent animals led to a lower FXR hepatic expression, avoiding its functional role of repressing BA accumulation in the liver [78]. Therefore, an accumulation of TBS in the liver appears after selenite supplementation. One of the key activities of FXR in the liver is downregulating the transcription of CYP7A1 in hepatocyte, the limiting enzyme which converts cholesterol into primary BAs. For this reason, hepatic BAs production is probably increased in S rats contributing to the higher TBS hepatic levels found in these animals. Moreover, FXR also inhibits MRP4, a BAs transporter expressed in the sinusoidal membrane of hepatocyte, contributing to blunting the sinusoidal secretion of BAs to blood [79]. However, in physiological states, this effect is overwhelmed by the induction of organic solute transporters (OST)αβ, two direct targets of FXR that contribute to secret BAs into the systemic circulation [80]. Low hepatic FXR activity, therefore, contributes to lower TBS serum systemic levels, as shown in S adolescent rats. Finally, FXR is essential for BA bile secretion, since it induces the expression of transporters at the biliary membrane of hepatocyte such as the BSEP (or ABCB11), which efflux BAs into the bile [81]. Even though TBS levels in bile have not been measured in this experiment, it is expected that, in S rats, this value was diminished. In fact, relative abundance in *Clostridium* clusters IV, XI and XIVa involved in bile acid 7α-dehydroxylation are decreased in S rats with respect to the rest of the group, indicating that a lower amount of primary TBS arrives in the colon [43]. Low selenite supplementation significantly decreased *Roseburia* (the most abundant in the colon of rats) and *Rombustia* populations, among others (Figure 3).

The repression of FXR found in the liver of S adolescent rats clearly contributes to the accumulation of TBS in the liver. To counteract this effect serum FGF21 levels are increased. The liver is considered the major source of the endocrine FGF21 that circulates in the blood and regulates energy expenditure and insulin sensitivity, favoring glucose consumption for heat production instead of energy storage [82]. Furthermore, FGF21 specifically acts in the liver, where it protects hepatocytes from metabolic stress caused by lipid overload. FGF21 stimulates hepatic fatty acid oxidation and reduces lipid flux into the liver by increasing peripheral lipoprotein catabolism and reducing adipocyte lipolysis [83]. Recently, different authors have revealed a previously unidentified role of FGF21 in BAs metabolism as a negative regulator of BAs synthesis [17]. The significantly higher levels of FGF21 found in S rats could be counteracting the higher BAs hepatic synthesis, impairing their accumulation in the liver.

Low SeNP supplementation in adolescent rats also led to an increase in TBS hepatic levels, but this increase was much more discrete than in selenite-supplemented rats. However, circulating serum TBS levels were unaltered, and the hepatokine FGF21 and the expression of the hepatic FXR suppressors of BAs synthesis was unaffected. In this context, the effects of low SeNP levels on TBS liver accumulation are lower than in S rats. This is in agreement with [10], who defend that SeNPs exhibit higher absorption in intestines due to their better bioavailability, having higher biological repercussion in this tissue than in metabolic tissue such as the liver compared to selenite. Since selenite is more retained and metabolized in the liver, it has more functional repercussions in this tissue, coinciding with the repletion in hepatic TBS and the significant changes in FXR function and FGF21 secretion. According to the more pronounced intestinal effects of SeNPs supplementation, low-SeNPs treatment reduced TBS enterohepatic circulation. The concentration of TBS in enterohepatic circulation is used as a marker of duodenal lipid absorption; therefore, lipid absorption could be affected after SeNPs treatment, contributing to lower WAT deposits [84]. The amount of TBS excreted by bile was not measured, but since the relative abundance of *Clostridium* involved in the bile acid 7α-dehydroxylation were not affected and the amount of TBS recaptured by enterohepatic circulation was decreased, the amount of TBS secreted in bile should be reduced. Moreover, the Gram-negative population and specifically *Bacteroides* are upregulated, as these kinds of bacteria are specifically less resistant to BAs, indicating that probably, intraluminal BAs are decreased [43,77]. Interestingly, microbiota not only produce secondary BAs, but also regulate their uptake and participate in the synthesis of primary BAs. Higher production of secondary BAs by microbiota activates ileum FXR activity in rats, which induces the production of FGF15 which is transported to the liver to activate hepatic FGF receptor 4 signaling to inhibit CYP7A1 gene transcription and primary BAs synthesis by an independent hepatic FXR function [68]. Maybe the lower amounts of intestinal BAs could be decreasing FGF15 secretion and collaborate to increase primary hepatic BAs synthesis without affecting hepatic FXR expression. The microbiota in the low-SeNP-treated rats seem to play a more important role than in the selenite group, since the changes are more relevant. In fact, [10] believes that gut microbiota plays a key role in SeNP conversion, a mechanism that remains unknown.

Finally, moderate-SeNPs therapy led to a general depletion in TBS pools since they are decreased in serum and enterohepatic circulation. Moreover, the downregulation of clostridia-relevant clusters to secondary BAs development indicated that the BA pool was also decreased in the intestine. However, hepatic TBS, FGF21 and FXR values were unaltered; however, colon microbiota turned into a pathogen profile where proteobacteria were increased, indicating that SeNPs mainly affect the gut due to their higher ability to be absorbed. This dose of SeNPs should be considered toxic, as we have described in previous papers [5].

Once again, low-SeNP treatment contributes to the decrease in lipid storage and the onset of obesity by reducing TBS enterohepatic circulation, which is clearly related to intestinal lipid absorption. In contrast, sodium selenite does not affect TBS enterohepatic circulation but increases hepatic TBS concentration, having a greater impact on liver metabolism by increasing FXR and FGF21 levels.

### 4.5. Contrasting Effects of Low Se Supplementation in the Colonic OTUS of Adolescent Rats in Relation to TBS and Lipid Homeostasis and Weight Management

**Figure 5:**
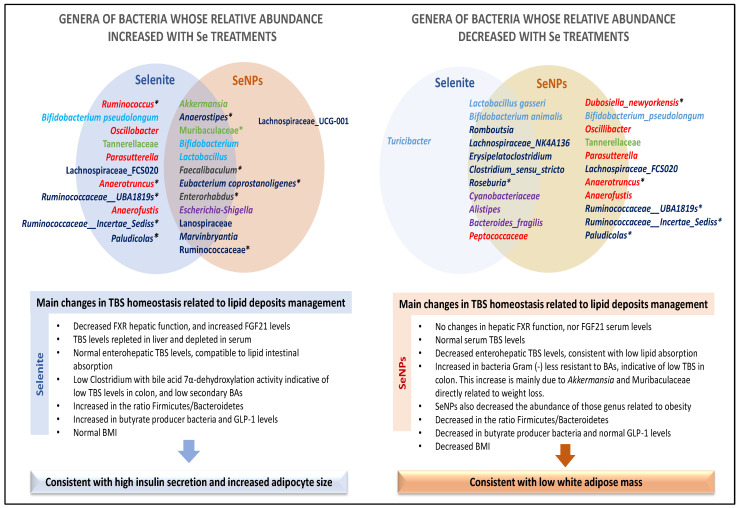
Genera of bacteria whose relative abundance changes with different Se treatments, and their functional repercussions. Main changes in TBS homeostasis related to lipid deposits management. Colours significance: (1) dark blue: bacteria with bile acid 7α-dehydroxylation activity; (2) light blue: bacteria with bile salt hydrolase activity; (3) green: bacteria associated with weight loss; (4) red: bacteria associated with obesity; and (5) purple: bacteria related to inflammation process. * Bacteria butyrate producer.

To recreate the main effects of low Se supplementation in the colonic microbiota of adolescent rats in relation to BAs and lipid homeostasis, all the OTUs detected were classified into five main functions. These functions were: weight management and lipid storage, butyrate production, bile salt hydrolase activity, bile acid 7α-dehydroxylation activity and those that link to dysbiosis and inflammation (Figure 5). Se administration in low doses, independently from its form of administration, increased two Gram-negative genus: *Akkermansia* and Muribaculaceae; both of them were clearly related to weight loss [85]. Their abundance was further enhanced in SeNP-treated rats. This tendency is in accordance with previous studies where SeNPs supplementation induced Akkermansia, Muribaculaceae, Bacteroides and Parabacteroides production that was related to beneficial lipidic profile by modulating the production of steroids [74]. Interestingly, *Akkermansia* has also been recently related to obesity resistance [85]. Moreover, relative to weight management and lipid storage, selenite supplementation led to a higher abundance of obesity-related bacteria, such as *Ruminococcus*, *Oscillibacter*, *Parasutterella*, *Anaerotruncus* and *Anaerofustis*, which affect adipose mass in rats [86]. Most of these genera are positively correlated with high-fat and low-fiber diets [59]. In particular, *Oscillibacter* has been proposed as a marker of obesity in adolescents [87]. In contrast, SeNPs treatment reduced the abundance of *Dubosiella newyorkensis*, *Oscillibacter*, *Parasutterella*, *Anaerotruncus* and *Anaerofustis*. Therefore, after selenite supplementation, there seems to be a balance among the abundance of microbiota related to weight loss and obesity, as finally, the BMI in these adolescent rats is unaltered. Nevertheless, after SeNP supplementation, the abundance of the genera related to weight loss was higher, together with those related to lipid storage and obesity lower. These results are in concordance with the lower BMI found in these rats and previous novel data which found a lower adipose mass and adipocyte size in adolescent SeNP-treated rats [5].

Proposed pathways by which microbiota could contribute to modifying lipid storage are the secretion of the incretin GLP-1 and the dysregulation of BAs signaling. Currently, they are both considered promising strategies for obesity therapy [85,88]. In this context, low Se administration, independently from its form of administration, increased the population of five butyrate producers: *Anaerostipes*, *Faecalibaculum*, *Eubacterium coprostanoligenes*, *Enterorhabdus* and Ruminococcaceae. However, selenite treatment led to a prominent effect on butyrate production by also increasing the abundance of *Ruminococcus*, *Anaerotruncus*, Ruminococcaceae *UBA1819*, Ruminococcaceae *Incertae Sedis* and *Paludicola*; the last four producers that were not detected in SeNP-treated rats. SCFAs regulate the proper function, motility and integrity of the GIT, and specifically, butyrate improves glucose homeostasis and strengthens satiety by increasing the production of GLP-1 in the intestine [70]. GLP-1 participates in glucose-stimulated insulin secretion from the pancreatic β-cells, improves insulin sensitivity and controls appetite. Moreover, butyrate helps to maintain the integrity of the gut epithelium via inducing mucin synthesis and improve the intestinal barrier by facilitating tight junction assembly. Finally, butyrate is the main energy source for colonocytes, and contributes to stimulating the immune system to properly respond to pathogens [89]. Consistently, as has been previously discussed, the serum GLP-1 values were increased in S animals, contributing to the higher insulin serum levels and WAT insulin sensitivity found in the previous studies on these animals [5].

Relative to the bacteria implicated in BAs homeostasis, three genera involved in the deconjugation of BAs showed an enhanced abundance after Se treatment in low doses: Muribaculaceae, *Bifidobacterium* and *Lactobacillus reuteri*; and two decreased: *Lactobacillus gasseri* and *Bifidobacterium animalis*. Selenite and SeNPs also decreased the different genera, but the total relative abundance of Gram-positive BSH bacteria seems to be compensated in both groups of animals, as is similar to those of control rats. However, the relative abundance of Muribaculaceae, the only Gram-negative bacteria with BSH activity and the third most abundant genus in a rat’s colon, was upregulated after SeNPs treatment. This fact indicates that, in this group of adolescent rats, there were more unconjugated BAs in the colon, which present less affinity to be transported by the enterohepatic circulation which is disposed of in the colon for bacteria with 7α-dehydroxylation activity [90]. The general effect of Se supplementation on the small population of *Clostridium* clusters IV, XI and XIVa encoding enzymes involved in bile acid 7α-dehydroxylation is clearly shown in Figure 3, and its relation to BS homeostasis is discussed. The decrease in the total relative abundance of these clusters, which only appears after selenite administration, is mainly due to a significant reduction in *Rombustia* (a Peptostreptococcaceae with great abundance in the colon of control rats) and *Roseburia*. They both have important 7α-dehydroxylation activity and are indicative of a reduction in secondary BA synthesis after selenite administration in the colon [41]. The composition of the BAs pool along the intestinal tract is closely associated with lipid absorption and storage, and perturbations of gut microbiota shape the BA composition. In this context, obesity has recently been related to lower *Clostridium scindens* abundance, which is directly correlated to a decrease in non-12-OH BAs and also, to an increase in *Turicibacter* and *Romboutsia*, which together, lower GLP-1 levels, and a decrease in hepatic FXR expression [85]. However, in this study, despite the selenite supplementation decreasing some genera of clostridia and hepatic FXR expression, the GLP-1 levels were enhanced, and *Romboutsia* and *Turicibacter* were decreased, as they were not compatible with obesity. In fact, selenite-treated rats had normal BMI and WAT deposits [5].

Finally, Se supplementation enhanced the abundance of the LPS producer *Escherichia-Shigella* abundance. Nevertheless, in general terms, this supplementation presents an anti-inflammatory function by decreasing the abundance of the pathogens Cyanobacteriaceae, *Alistipes* and *Bacteroides fragilis*, particularly in SeNP administration. The trace element Se, through its incorporation into selenoproteins, plays an important role in oxidative balance, inflammation and immunity. Adequate levels of Se are important for initiating immunity and regulating excessive immune responses and chronic inflammation [91]. Particularly, in this study, Se administration in the form of SeNPs at physiological levels seems to be even more effective in regulating inflammatory processes. Consistent with these results, several authors have described the specific anti-inflammatory effects of SeNPs supplementation on the intestinal tract and systemic circulation. Supranutritional SeNPs contribute to alleviated intestinal oxidative stress and intestinal barrier dysfunction [74]. The role of SeNPs in pharmacological protection against various inflammatory and oxidative stress-mediated conditions is also known, mainly by the modification of signaling proteins, such as the transcription factor NFkB [92]. For instance, in a previous paper using the same adolescent rats and Se treatments that are in this current study, the main pro-inflammatory cytokine (TNF-α) regulated by NFkB was reduced after Se treatment in serum, but especially after SeNPs administration [5]. This study also described a specific role of SeNPs administration by increasing FOXO3a autophagy pathway related to lower inflammation.

Since some colonic microbiota populations are clearly related to both being lean and obesity, and the proposed pathways by which microbiota could modify lipid storage include the secretion of GLP-1 and the dysregulation of BA signaling, low SeNP supplementation during adolescence is a clear candidate to prevent obesity. It increases the abundance of genera related to weight loss and reduces those related to lipid storage and obesity induction. Additionally, it affects TBS homeostasis, leading to a reduction in lipid intestinal absorption. Conversely, sodium selenite supplementation increases the abundance of populations related to butyrate and GLP-1 induction and affects TBS homeostasis mainly in the liver. It modulates liver metabolism, induces insulin signaling and promotes lipid anabolism without increasing BMI. Therefore, it is a potential supplementation to consider during the cachexia process in adolescence related to drug consumption or anorexia.

## 5. Conclusions

Adolescence is a period when body composition changes deeply; low-Se treatment of adolescent rats in the form of soluble selenite or SeNPs has contrasting effects on their lipid adipose mass. In particular, soluble selenite administration leads to higher adipocytes size and hyperinsulinemia without obesity and normal BMI, and SeNPs treatment significantly decreased lipid deposits consistently with autophagy, hipoinsulemia and low BMI. However, these effects involve a network of correlated processes in which colonic microbiota contributes to modifying lipid storage by the secretion of the incretin GLP-1, and by the regulation of TBS homeostasis, necessary for lipid intestinal absorption. In fact, the administration of selenite or SeNPs produces contrasting changes in the microbiota of adolescent rats; these changes are greater after the better intestinal absorbed form of SeNPs. SeNPs affected microbiota towards a more prominent Gram-negative profile in which the relative abundance of the bacteria related to weight loss *Akkermansia* and Muribaculaceae were clearly enhanced, and the weight gain ratio F/B reduced. This bacterial profile together with a lower abundance of the obesity inducer *Dubosiella newyorkensis*, *Oscillibacter*, *Parasutterella*, *Anaerotruncus* and *Anaerofustis* are directly related to lower adipose tissue mass. SeNP administration also modifies TBS homeostasis, mainly by decreasing their concentration in enterohepatic circulation, indicating lower intestinal lipid absorption. In contrast, selenite administration mainly affects liver function by decreasing FXR hepatic function, leading to the accumulation of hepatic TBS, which was decreased in serum. This supplementation also impacts colonic microbiota, it has a prominent effect on butyrate production by increasing the abundance of the population of 10 known butyrate producers increasing GLP-1 secretion. Our results suggest the possibility of regulating WAT and BMI upon the administration of different forms of Se, whereas selenite-treated rats show a tendency to anabolism; those treated with SeNPs show a tendency towards leanness, and these responses are apparently modulated by the changes in the microbiota that correlate with BAs metabolism, butyrate production, insulin signaling and inflammation. These actions have potential implications for the management of obesity and anorexia during adolescence. In this context, future pre-clinical studies should be undertaken, taking into account obese and cachexia rat models.

## Figures and Tables

**Table 1:** Nutritional and duodenum parameters at the end of the experimental period.

	C	S	NS	NSS
Increased body weight (g/d)	6.01 ± 0.1	6.07 ± 0.2	5.98 ± 0.2	5.13 ± 0.2 *^,s^
Solid intake (g/d)	18.2 ± 0.3	18.6 ± 0.2	18.3 ± 0.1	16.5 ± 0.2 ***^,sss,ppp^
Liquid intake (mL/d)	21.4 ± 0.7	21.5 ± 0.7	19.2 ± 0.8	16.7 ± 0.5 ***^,sss,pp^
Total Se intake (µg/d)	3.48 ± 0.08	6.81 ± 0.14 **	6.59 ± 0.09 **	31.35 ± 1.43 ***^,sss,ppp^
Duodenum weight (mg/cm^2^)	103 ± 2.7	99 ± 2.9	93.2 ± 3.8	83 ± 3.6 **^,s^
Mucosa weight (mg/cm^2^)	38.2 ± 1.1	35 ± 1.5	41 ± 1.1 ^s^	44 ± 1.8 *^,ss^
Duodenum perimeter (cm)	0.94 ± 0.06	0.97 ± 0.04	1.02 ± 0.01 *	1.01 ± 0.01 *
EGF (pg/mL)	9.8 ± 0.19	21.9 ± 0.5 ***	5.8 ± 0.09 **^,sss^	3.1 ± 0.16 ***^,sss,p^

The mean ± SEM values of the results were analysed using a multifactorial one-way ANOVA and Tukey’s test. Groups label: C (control), S (low-sodium selenite), NS (low-SeNPs) and NSS (moderate-SeNPs). Epidermal Growth Factor (EGF). Significance: vs. C, * *p* < 0.05, ** *p* < 0.01, *** *p* < 0.001; vs. S, ^s^ *p* < 0.05, ^ss^ *p* < 0.01, ^sss^ *p* < 0.001; vs. NP, ^p^ *p* < 0.05, ^pp^ *p* < 0.01, ^ppp^ *p* < 0.001.

**Table 2:** Mean relative abundance of main phyla of Gram-positive and Gram-negative and values of lipopolysaccharides (LPS) in serum.

	C	S	NS	NSS
Relative abundance of Phyla Gram-positive (%) (Firmicutes + Actinobacteriota)	75.9 ± 3.5	70 ± 2.9	62 ± 3.1 *	73.4 ± 3.5
Relative abundance of Phyla Gram-negative (%) (Verrucomicrobiota + Bacteroidota + Proteobacteria)	23.8 ± 1.1	29.8 ± 1.7	37.9 ± 2.9 **	26.5 ± 1.5
Serum LPS (pg/mL)	0.49 ± 0.01	0.57 ± 0.02	0.62 ± 0.04 *	0.59 ± 0.03

The mean ± SEM values of the results were analysed using a multifactorial one-way ANOVA and Tukey’s test. Groups label: C (control), S (low-sodium selenite), NS (low SeNPs) and NSS (moderate-SeNPs). Significance: vs. C, * *p* < 0.05, ** *p* < 0.01.

**Table 3:** Summary of OTUs whose relative abundance increased in both S and NS groups, related to OTUs main roles. OTUs in table are ordered by their relative abundance in control rats.

OTU	Folds Inductions		Characteristics or Comments on These OTUs	References
	S	NS		
*Akkermansia*	1.4	1.6	Proposed as probiotic for weight loss.	[39,40]
*Anaerostipes*	1.3	2.4	Involved in bile acid 7α-dehydroxylation and Butyrate producer.	[41,42]
Muribaculaceae	1.8	5.4	The only Gram (-) bacteria with bile salt hydrolase activity. Related to weight loss.	[43,44]
*Bifidobacterium*	2.5	2.2	Probiotic with bile salt hydrolase activity.	[43]
*Lactobacillus reuteri*	1.7	2.6	Probiotic with bile salt hydrolase activity.	[43]
*Faecalibaculum*	3.2	1.9	Butyrate producer.	[42]
*Eubacterium_coprostanoligenes*	1.8	1.5	Involved in bile acid 7α-dehydroxylation and Butyrate producer.	[41,42]
*Enterorhabdus*	2.6	2.4	Indirect Butyrate producer.	[45]
*Enterorhabdus mucosicola*	3.4	2.6	Indirect Butyrate producer. Regulates glucose uptake in adipocytes via insulin.	[46]
*Escherichia-Shigella*	1.5	2.2	Lipopolysaccharide producer.	[47]
Lachnospiraceae	1.4	1.6	Involved in bile acid 7α-dehydroxylation.	[41]
*Marvinbryantia*	5.1	4.3	Involved in bile acid 7α-dehydroxylation.	[41]
Ruminococcaceae	6.7	4.0	Butyrate producer.	[42]

**Table 4:** Summary of OTUs whose relative abundance decreased in both S and NS groups, related to OTUs main roles. OTUs in table are ordered by their relative abundance in control rats.

OTU	Folds Reduction		Characteristics or Comments on These OTUs	References
	S	NS		
*Lactobacillus gasseri*	0.87	0.87	Probiotic with bile salt hydrolase activity.	[43]
*Romboutsia*	0.66	0.80	Involved in bile acid 7α-dehydroxylation. It has bile salt hydrolase and urease enzymes.	[41,48]
*Bifidobacterium animalis*	0.55	0.21	Probiotic with bile salt hydrolase activity.	[43]
Lachnospiraceae *NK4A136*	0.78	0.38	Involved in bile acid 7α-dehydroxylation.	[41]
*Erysipelatoclostridium*	0.42	0.28	Involved in bile acid 7α-dehydroxylation.	[41]
*Clostridium sensu stricto*	None	None	Involved in bile acid 7α-dehydroxylation.	[41]
*Roseburia*	0.08	0.37	Involved in bile acid 7α-dehydroxylation and Butyrate producer.	[41,42]
Cyanobacteriaceae	0.55	0.22	Associated with disease, not only in the GIT but also to neurological diseases, lung cancer and rhinitis allergy.	[49]
*Alistipes*	0.48	0.02	Related to gut inflammation, colorectal cancer and neurological disorders.	[50]
*Bacteroides fragilis*	0.12	0.05	It is a gut commensal which can be the cause of multiple peritoneal, vaginal or urinary infections, septicaemia, etc.	[51]
Peptococcaceae	0.73	0.31	Directly related to obesity and insulin resistance.	[52]

**Table 5:** Summary of OTUs whose relative abundance was contrasting in both S and NS groups, related to OTUs main roles. OTUs in table are ordered by their relative abundance in control rats.

OTU	Folds Changes		Characteristics or Comments on These OTUs	References
	S	NS		
*Turicibacter*	0.41	1.06	It has bile salt hydrolase activity.	[43]
*Dubosiella newyorkensis*	1.02	0.31	Correlated with body weight gain and obesity. Involved in bile acid 7α-dehydroxylation.	[41,53]
*Ruminococcus*	3.00	0.91	Involved in bile acid 7α-dehydroxylation and Butyrate producer. Associated with obesity and GDM.	[41,42,54,55]
*Bifidobacterium pseudolongum*	2.39	0.70	Probiotic with bile salt hydrolase activity.	[43]
*Oscillibacter*	2.61	0.44	Inversely related to weight loss. Involved in bile acid 7α-dehydroxylation.	[41,56]
Lachnospiraceae *UCG001*	0.98	2.21	Involved in bile acid 7α-dehydroxylation.	[41]
Tannerellaceae	1.59	0.12	Considered a marker of healthy/lean mice.	[57]
*Parasutterella*	1.87	0.25	Correlated with BMI and DMTII.	[58]
Lachnospiraceae *FCS020*	1.90	0.19	Involved in bile acid 7α-dehydroxylation.	[41]
*Anaerotruncus*	4.26	0	Involved in bile acid 7α-dehydroxylation and Butyrate producer. It is associated with obesity.	[41,42,59]
Ruminococcaceae *UBA1819*	1.48	0	Involved in bile acid 7α-dehydroxylation and Butyrate producer.	[41,42].
*Anaerofustis*	2.92	0.55	Involved in bile acid 7α-dehydroxylation. Its diminution correlates with weight loss.	[41,60]
Ruminococcaceae *Incertae Sedis*	3.00	0	Involved in bile acid 7α-dehydroxylation and Butyrate producer.	[41,42]
*Paludicola*	1.38	0	Involved in bile acid 7α-dehydroxylation and Butyrate producer.	[41,42]

## Data Availability

Not applicable.
